# Antibody–Drug Conjugates in Oncology: Principles, Clinical Development, and Future Directions

**DOI:** 10.1002/mco2.70699

**Published:** 2026-03-30

**Authors:** Bisheng Cheng, Lanqi Gong, Zongwei Wang, Peidan Peng, Kewei Xu, Hai Huang, Peng Wu

**Affiliations:** ^1^ Department of Urology Nanfang Hospital Southern Medical University Guangzhou China; ^2^ Department of Surgery Division of Urology, Beth Israel Deaconess Medical Center Harvard Medical School Boston Massachusetts USA; ^3^ Department of Clinical Oncology, Li Ka Shing Faculty of Medicine The University of Hong Kong Hong Kong, SAR China; ^4^ Department of Clinical Oncology The University of Hong Kong‐Shenzhen Hospital Shenzhen China; ^5^ Department of Urology Sun Yat‐Sen Memorial Hospital, Sun Yat‐Sen University Guangzhou China; ^6^ Southern Medical University Guangzhou China

**Keywords:** antibody–drug conjugates, drug resistance, therapeutic index, tumor microenvironment

## Abstract

Antibody–drug conjugates (ADCs) have emerged as a major therapeutic modality in oncology, enabling the targeted delivery of highly potent cytotoxic agents while expanding the therapeutic window in solid tumors. Recent clinical successes across breast, lung, and genitourinary cancers have highlighted that ADC efficacy is governed not only by target expression, but also by the integrated optimization of antibody engineering, linker chemistry, payload selection, and tumor‐specific biology. In this review, we summarize the fundamental principles underpinning ADC design, including antibody format and Fc engineering, linker stability, payload classes, drug‐to‐antibody ratio optimization, and the bystander effect. We then discuss tumor antigen biology and target landscapes across solid tumors, with particular emphasis on how antigen density, heterogeneity, internalization kinetics, and intracellular trafficking shape clinical activity. Uro‐oncological malignancies—especially urothelial carcinoma—are presented as a clinically advanced and instructive paradigm for ADC development. Experience from these tumors illustrates both the opportunities and limitations of ADC therapy, including mechanisms of response and resistance, biomarker‐driven patient selection, rational combination strategies, and safety management in real‐world practice. Finally, we provide a forward‐looking perspective on next‐generation ADC development, highlighting emerging conjugation technologies, bispecific and conditionally activated ADCs, strategies to overcome resistance, and evolving clinical trial designs. By integrating engineering principles with tumor biology and clinical execution, this review aims to offer a translational framework to guide the future development and implementation of ADCs across oncology.

## Graphical abstract

Antibody–drug conjugates integrate antibody targeting with potent cytotoxic payloads to enable precision cancer therapy. Their clinical performance depends on coordinated optimization of engineering design, tumor antigen biology, and treatment strategy. Lessons from uro‐oncological malignancies illustrate how ADCs can be effectively developed, combined, and implemented, informing next‐generation ADC innovation across solid tumors.

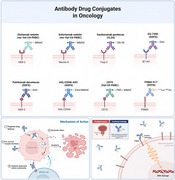

## 1 Introduction

Antibody–drug conjugates (ADCs) have rapidly evolved from a conceptual “magic bullet” into one of the most influential therapeutic platforms in modern oncology. By coupling the target specificity of monoclonal antibodies with the cytotoxic potency of small‐molecule payloads, ADCs offer a unique strategy to expand the therapeutic window of cancer treatment, particularly in solid tumors where conventional chemotherapy and targeted agents often face limitations related to toxicity, resistance, and tumor heterogeneity [1, 2, 3].

Over the past decade, technological advances in antibody engineering, linker chemistry, and payload design have fundamentally reshaped the clinical landscape of ADCs. Early generations of ADCs were constrained by unstable linkers, heterogeneous conjugation, and narrow therapeutic indices, leading to inconsistent efficacy and unacceptable toxicity. In contrast, contemporary ADCs demonstrate that clinical performance is determined by the coordinated optimization of multiple interdependent components, including antigen selection, internalization kinetics, intracellular trafficking, payload mechanism of action, and drug‐to‐antibody ratio [4, 5]. These insights have enabled ADCs to achieve clinically meaningful survival benefits across several solid tumors, including breast, lung, and genitourinary cancers [6, 7].

Importantly, ADC efficacy is not dictated by target expression alone. Accumulating clinical and translational evidence indicates that tumor architecture, antigen heterogeneity, lysosomal competence, and microenvironmental constraints critically shape payload delivery and response durability [8, 9]. As ADCs are increasingly deployed in earlier lines of therapy and in combination with immune checkpoint inhibitors or other targeted agents, a more integrated understanding of engineering principles, tumor biology, and clinical implementation has become essential [10, 11].

Within this broader oncological context, uro‐oncological malignancies have emerged as a particularly instructive setting for ADC development. Urothelial carcinoma (UC) has become one of the most clinically advanced disease models for ADC therapy, whereas prostate and renal cancers highlight distinct biological and translational constraints. Rather than serving as a narrow disease‐specific focus, these malignancies collectively provide a representative paradigm through which the opportunities, limitations, and future directions of ADC therapy across solid tumors can be critically examined [12, 13, 14].

In this review, we synthesize current understandings of ADC engineering and tumor antigen biology, examine clinical development and resistance mechanisms with an emphasis on lessons from uro‐oncology, and discuss emerging strategies that are likely to shape the next generation of ADCs. By integrating fundamental principles with disease‐specific insights, we aim to provide a translational framework to guide the rational development and clinical implementation of ADCs across oncology.

## 2 Fundamental Principles of ADC Design and Engineering

**FIGURE 1:**
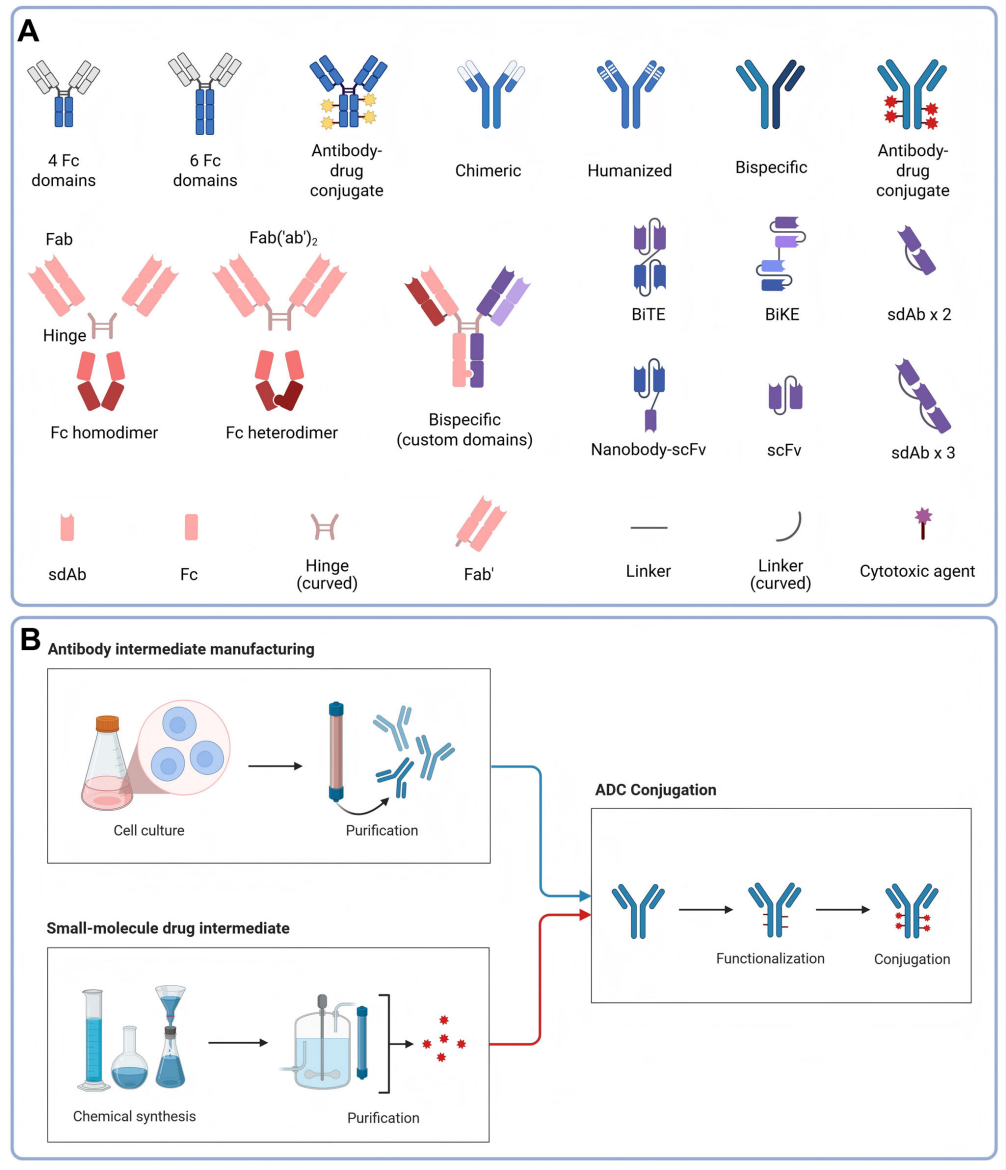
Modular design and assembly of antibody–drug conjugates. (A) Schematic overview of the modular architecture of antibody–drug conjugates (ADCs), illustrating representative antibody formats (full‐length IgG, antibody fragments, and engineered bispecific constructs), linker strategies, and cytotoxic payload classes that can be combined to generate distinct ADC platforms. (B) General ADC assembly workflow, in which antibody and payload intermediates are independently manufactured and subsequently conjugated using defined chemistries, highlighting how variations in antibody format, linker chemistry, and payload selection collectively shape ADC pharmacology, efficacy, and safety. ADC, antibody–drug conjugate; F(‘ab’)_2_, bivalent fragment antigen‐binding; Fab, fragment antigen‐binding; Fc, fragment crystallizable; IgG, immunoglobulin G; scFv, single‐chain variable fragment; sdAb, single‐domain antibody.

The clinical success of ADCs depends not on any single component, but on the integrated optimization of antibody architecture, linker chemistry, and payload properties. Advances across solid tumors have demonstrated that subtle engineering decisions can profoundly influence pharmacokinetics, tumor penetration, intracellular payload release, and toxicity profiles. Understanding these principles is therefore foundational to rational ADC development [15, 16] (Figure 1).

### 2.1 Antibody Format and Fc Engineering

Most clinically approved ADCs are based on an IgG1 backbone, which retains the capacity to engage Fcγ receptors and mediate antibody‐dependent cellular cytotoxicity or phagocytosis. In certain tumor contexts, these Fc‐mediated immune effector functions may synergize with payload‐induced immunogenic cell death, amplifying antitumor activity beyond direct cytotoxicity alone. However, Fc engagement can also contribute to off‐tumor uptake and systemic clearance, necessitating careful balancing of immune activation and pharmacokinetic stability [17].

To address these challenges, multiple antibody engineering strategies have been explored. Fc silencing can reduce non‐specific immune engagement, whereas afucosylation enhances FcγRIIIa binding and antibody‐dependent cellular cytotoxicity [18]. Biparatopic antibodies, which recognize two nonoverlapping epitopes on the same antigen, can promote receptor clustering and accelerate internalization [19]. More recently, bispecific ADC formats have been developed to improve tumor selectivity or to exploit alternative endocytic pathways, foreshadowing a new generation of multifunctional ADC platforms [20].

### 2.2 Linker Chemistry and Systemic Stability

The linker is a critical determinant of ADC safety and efficacy, governing both plasma stability and intracellular payload release 21. Cleavable linkers, such as valine‐citrulline‐based systems, are designed to exploit tumor‐associated lysosomal protease activity, enabling efficient payload liberation and bystander killing. In contrast, non‐cleavable linkers rely on complete lysosomal degradation of the antibody component, generating charged metabolites with limited membrane permeability and reduced bystander effect [22].

Although cleavable linkers can enhance efficacy in antigen‐heterogeneous tumors, they also increase the risk of systemic payload release if plasma stability is compromised. Even minor chemical modifications can significantly alter linker susceptibility to off‐target cleavage, underscoring the importance of precise linker design tailored to both payload chemistry and tumor biology.

### 2.3 Payload Classes and Mechanisms of Action

The payload ultimately determines the cytotoxic potency of an ADC and shapes its toxicity profile. Microtubule inhibitors, such as auristatins, remain widely used due to their high potency but are associated with characteristic adverse events, including peripheral neuropathy and dermatologic toxicity. Topoisomerase I inhibitors induce DNA damage and replication stress, often triggering immunogenic cell death and providing a strong biological rationale for combination with immune checkpoint blockade [23, 24, 25].

More potent payloads, including pyrrolobenzodiazepine dimers and duocarmycins, offer the theoretical advantage of activity in low‐antigen settings but are frequently limited by narrow therapeutic windows. Their safe clinical deployment increasingly depends on sophisticated linker strategies and controlled payload release, highlighting the inseparability of payload selection and conjugation chemistry.

### 2.4 Drug‐to‐Antibody Ratio Optimization and Controlled Release

Increasing the drug‐to‐antibody ratio can enhance payload delivery per binding event, but excessively high ratios often accelerate clearance, promote aggregation, and exacerbate toxicity. Site‐specific conjugation technologies have therefore been developed to generate homogeneous ADCs with defined drug loading, improving pharmacokinetic predictability and manufacturability [26].

Beyond static optimization, next‐generation platforms aim to achieve spatially and temporally controlled payload release. Tandem linkers, conditionally activated ADCs, and tumor‐restricted pro‐drug designs seek to decouple systemic exposure from intratumoral potency, offering a promising strategy to widen the therapeutic index of highly potent warheads.

### 2.5 The Bystander Effect and Tumor Penetration

The bystander effect represents a double‐edged sword in solid tumor ADC therapy. Membrane‐permeable payloads released from antigen‐positive cells can eradicate neighboring antigen‐negative tumor populations, overcoming spatial heterogeneity that characterizes many epithelial malignancies. However, this same property can mediate collateral damage to normal tissues with low‐level antigen expression, contributing to dose‐limiting toxicity [27].

Physical barriers within the tumor microenvironment, including abnormal vasculature, dense stroma, and hypoxic niches, further restrict ADC distribution [28, 29]. Engineering strategies to enhance tumor penetration must therefore balance improved payload delivery against systemic tolerability, reinforcing the need for holistic ADC design.

## 3 Tumor Antigen Biology and Target Landscapes Across Solid Tumors

The biological suitability of a tumor antigen is a central determinant of ADC efficacy [30, 31]. Unlike small‐molecule targeted therapies, where intracellular kinase inhibition may be achieved with minimal receptor occupancy, ADCs rely on a multistep process encompassing surface binding, receptor‐mediated internalization, intracellular trafficking, lysosomal processing, and payload release [32]. As a result, antigen prevalence alone is insufficient to predict clinical activity. Instead, a constellation of biological features—including antigen density, spatial heterogeneity, internalization kinetics, and tolerable expression in normal tissues—collectively defines whether an antigen is functionally actionable for ADC therapy 33.

**FIGURE 2:**
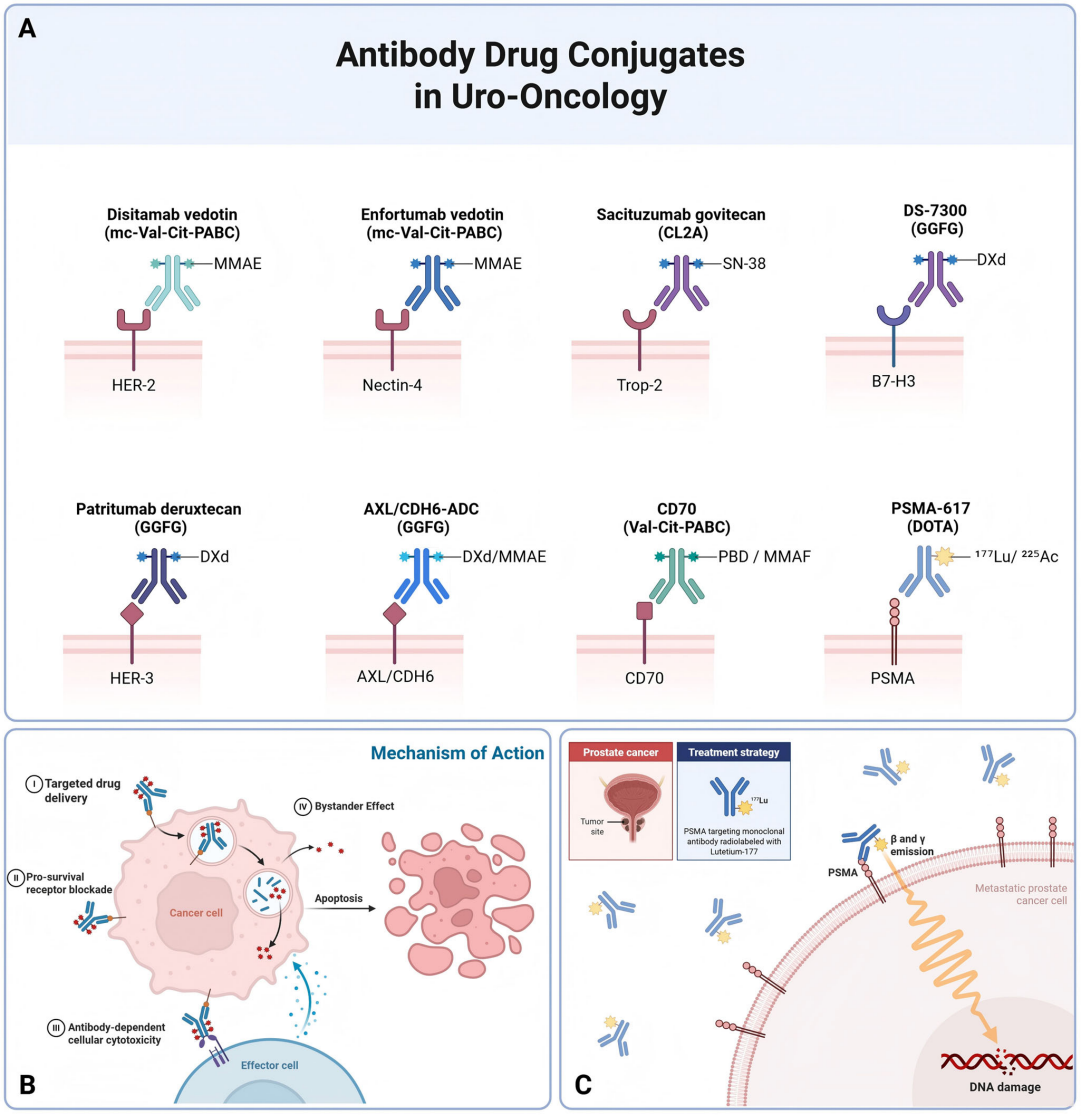
ADC and radioligand therapeutic strategies in uro‐oncology. (A) Representative target‐linker‐payload architectures of clinically advanced or late‐stage investigational ADCs in urothelial and prostate cancers, illustrating the diversity of antigen selection and conjugation chemistry. (B) Canonical and accessory mechanisms of ADC action following antigen binding and internalization, including lysosomal payload release, microtubule disruption or DNA damage, Fc‐mediated effector functions, and bystander killing that can overcome antigen heterogeneity. (C) Conceptual comparison with PSMA radioligand therapy, in which low‐molecular‐weight ligands deliver β‐ or α‐emitting radionuclides to metastatic lesions, inducing clustered DNA damage independent of intracellular payload release. ADCC, antibody‐dependent cellular cytotoxicity; DAR, drug‐antibody ratio; DXd, deruxtecan; MMAE/F, monomethyl auristatin E/F; PBD, pyrrolobenzodiazepine; PSMA, prostate‐specific membrane antigen; SN‐38, active irinotecan metabolite.

Across solid tumors, the expanding clinical experience with ADCs has refined our understanding of what constitutes an effective target. Importantly, these principles are broadly applicable across tumor types, yet are particularly well illustrated in uro‐oncological malignancies, which span a wide spectrum of epithelial differentiation states, antigen heterogeneity, and tumor microenvironmental constraints [34, 35]. In this section, we discuss general determinants of ADC target suitability and then examine representative antigen classes across solid tumors, highlighting how lessons from uro‐oncology inform pan‐oncologic ADC development 36 (Figure 2).

### 3.1 Determinants of an Effective ADC Target

An ideal ADC target exhibits high and homogeneous expression on tumor cells, limited accessibility in vital normal tissues, and efficient internalization upon antibody engagement. However, few clinically relevant antigens fulfill all of these criteria simultaneously. Clinical experience has therefore shifted the emphasis from identifying “perfect” targets to understanding how engineering strategies can compensate for biological imperfections.

Antigen density influences the number of ADC molecules delivered per cell, but its predictive value is nonlinear. tumors with moderate antigen expression may remain sensitive if internalization is rapid and payload release is efficient, whereas tumors with high surface expression may be resistant if intracellular trafficking is unproductive. Moreover, target‐mediated drug disposition can accelerate systemic clearance when antigens are abundantly expressed in normal tissues or shed into circulation, narrowing the therapeutic window 37.

Internalization kinetics and intracellular routing are equally critical. Productive ADC activity requires trafficking to lysosomal compartments capable of degrading the antibody or cleaving the linker. Antigens that preferentially recycle to the cell surface or divert to non‐lysosomal pathways can substantially attenuate payload delivery, even in the presence of preserved binding affinity. These considerations highlight why ADC target biology must be evaluated as a dynamic, functional process rather than a static expression readout.

### 3.2 Antigen Heterogeneity and Spatial Tumor Biology

Intratumoral heterogeneity represents one of the most formidable barriers to ADC efficacy in solid tumors. Spatially variable antigen expression, driven by clonal evolution, epithelial‐mesenchymal transition, lineage plasticity, and treatment‐induced selection, can create mixed populations of antigen‐positive and antigen‐negative tumor cells within the same lesion. This phenomenon is particularly pronounced in advanced epithelial malignancies and contributes to both primary resistance and relapse following initial ADC response [38].

The bystander effect partially mitigates this challenge by enabling membrane‐permeable payloads to diffuse from antigen‐positive cells into neighboring antigen‐negative cells. However, the extent of bystander killing is constrained by payload chemistry, local diffusion barriers, and stromal architecture 39. Dense extracellular matrix, abnormal vasculature, and hypoxic niches can further restrict ADC penetration and payload distribution, resulting in spatially heterogeneous drug exposure.

These considerations underscore the importance of spatial tumor biology in ADC development. Quantitative and spatial assessment of antigen expression—rather than binary positivity—has therefore become increasingly relevant for patient selection and response prediction, particularly in tumors characterized by marked architectural complexity 40, 41.

### 3.3 Canonical ADC Targets Across Solid Tumors

Several antigen classes have emerged as broadly relevant ADC targets across solid tumors, each exemplifying distinct biological trade‐offs between tumor specificity, heterogeneity, and payload dependency.

HER2 represents a paradigmatic example of how ADC engineering can redefine target biology. Although HER2 amplification historically guided patient selection for monoclonal antibodies and kinase inhibitors, HER2‐directed ADCs have demonstrated activity across a wider expression spectrum, including so‐called HER2‐low tumors [42]. This expanded therapeutic scope reflects the contribution of bystander killing and highlights how ADCs can exploit antigen accessibility and intracellular processing rather than absolute overexpression.

TROP2 is another widely expressed epithelial antigen that illustrates a different targeting paradigm [43]. Its expression is often continuous rather than binary, and its utility as an ADC target derives largely from efficient internalization and payload‐driven cytotoxicity rather than tumor‐restricted expression. As a result, TROP2‐directed ADC efficacy is frequently decoupled from high antigen density, shifting the emphasis toward downstream determinants such as DNA damage response capacity and host pharmacogenomics [44].

Nectin‐4 exemplifies a target with favorable tumor enrichment and internalization properties, particularly in UC. Although its expression is heterogeneous and dynamically regulated, the combination of efficient lysosomal trafficking and membrane‐permeable payloads has enabled robust clinical activity 45. Nectin‐4 thus illustrates how functional target biology can partially compensate for spatial heterogeneity, while also defining clear boundaries of sensitivity when antigen loss becomes profound.

### 3.4 Uro‐Oncology as a Representative Model for ADC Target Biology

Uro‐oncological malignancies provide a uniquely instructive framework for understanding ADC target biology across solid tumors. UC, prostate cancer, and renal cell carcinoma (RCC) collectively encompass a broad range of antigen landscapes, from highly epithelial and permissive to lineage‐restricted and biologically constrained 46.

UC is characterized by relatively high epithelial antigen expression and permissive tumor architecture, conditions that favor ADC efficacy when coupled with appropriate payload and linker design. In contrast, prostate cancer highlights the challenges of lineage specificity and phenotypic plasticity. Although several prostate‐associated antigens exhibit high tumor specificity, their heterogeneous expression and dynamic regulation under androgen deprivation can limit durable ADC responses. RCC presents yet another paradigm, where hypoxia‐driven transcriptional programs generate lineage‐enriched targets, but abundant vascularization and metabolic adaptability complicate intracellular payload delivery [47].

Together, these malignancies illustrate that antigen prevalence alone does not dictate ADC success. Instead, the interaction between antigen biology, tumor architecture, intracellular trafficking, and payload properties determines whether a given target is clinically actionable. The diversity of uro‐oncological antigen landscapes therefore offers transferable lessons for ADC development in other solid tumors, informing target prioritization, engineering strategy, and biomarker integration [48].

### 3.5 Implications for Next‐Generation ADC Target Selection

The evolving understanding of tumor antigen biology has important implications for next‐generation ADC development. Target selection is increasingly viewed as an iterative process that integrates biological characterization with engineering feasibility and clinical context. Rather than excluding imperfect targets, contemporary strategies seek to tailor ADC design—through linker choice, payload permeability, and controlled release mechanisms—to overcome inherent biological constraints.

Looking forward, advances in spatial profiling, functional imaging, and single‐cell analysis are expected to further refine target evaluation by capturing dynamic changes in antigen expression and intracellular processing. These approaches will be essential for aligning ADC engineering with tumor biology and for extending the clinical reach of ADCs beyond traditionally “ideal” targets.

## 4 Clinical Development of ADCs: Lessons From Uro‐Oncological Malignancies

The translation of ADCs from conceptual platforms to clinically transformative therapies has been most clearly realized in uro‐oncological malignancies. Among solid tumors, UC has emerged as a paradigm‐setting disease for ADC development, whereas prostate and renal cancers illustrate distinct biological and translational constraints. Together, these malignancies provide a clinically grounded framework for understanding how antigen biology, ADC engineering, and treatment context converge to determine therapeutic success or failure [49].

Rather than serving as isolated disease‐specific examples, the uro‐oncology experience offers transferable lessons that inform ADC development across oncology, particularly with respect to patient selection, sequencing strategies, and integration into evolving treatment paradigms [50] (Table 1).

**TABLE 1:** Selected pivotal and representative recent antibody–drug conjugate (ADC) clinical trials in uro‐oncology.

Agent (target/linker‐payload)	Setting and line	Population/Biomarker	Design/Comparator	Primary endpoints	Key efficacy signals	Dominant toxicities	Construct notes	Development status	Source/Registry
Enfortumab vedotin + Pembrolizumab (Nectin‑4/MMAE)	1L la/mUC	All‑comers; no antigen test	Randomized phase 3 vs. gemcitabine + platinum	OS, PFS	OS↑, PFS↑; new 1L standard	Rash, hyperglycemia, PN	Cleavable Val‑Cit; bystander capable	Approved (combo) in many regions	[51]
Enfortumab vedotin (monotherapy)	Post‑platinum/Post‑IO	Heavily pretreated	Single‑arm pivotal	ORR	ORR moderate‑high; DOR ∼7–8 months	Rash, PN, fatigue	Same construct	Historical later‑line approval basis	[52]
Sacituzumab govitecan (Trop‑2/SN‑38)	≥2 L la/mUC	Trop‑2 not required	Single‑arm phase II (key cohorts)	ORR (key), PFS, OS	Durable responses; after‑EV activity variable	Neutropenia, diarrhea, alopecia	Topo‑I payload with strong bystander effect	Randomized programs ongoing/region‑specific approvals	[53]
Disitamab vedotin ± PD‑1 (HER2/MMAE)	1L la/mUC (HER2‑expressing)	IHC 1+–3+ (HER2‑low included)	Randomized phase 3 vs. chemo	PFS (key), OS	Signals across HER2 strata	Dermatologic, PN; ILD vigilance	Cleavable MMAE; bystander effect	Phase III + RWD; combos active	[54]
Ifinatamab deruxtecan (DS‑7300; B7‑H3/DXd)	Later‑line la/mUC	B7‑H3 broadly expressed	Basket/phase I‐II	ORR, PFS	Preliminary activity	Cytopenias; ILD risk	DXd bystander payload	Expansion cohorts ongoing	[55]
Tisotumab vedotin (tissue factor/MMAE)	Exploratory in UC	TF variably expressed	Basket/phase I‐II	ORR	Mixed activity; ocular prophylaxis needed	Ocular events, epistaxis, PN	Cleavable MMAE	Signal‑seeking only	[56]
FOR46 (CD46/MMAE)	mCRPC post‑ARPI ± chemo	CD46 high; no companion Dx	Phase I/II single‑arm; combo cohorts	ORR/PSA50, safety	Early responses incl. soft‑tissue disease	Neuropathy, cytopenias	Cleavable MMAE	Active; combinations with ARPI	[57]
Ifinatamab deruxtecan (DS‑7300; B7‑H3/DXd)	mCRPC (multi‑line)	B7‑H3 high in CRPC/AR‑indifferent	Basket/phase I‐II	ORR, rPFS	Promising multi‑tumor signals	Cytopenias; ILD risk	DXd bystander	PCa cohorts ongoing	[58]
PSMA‑ADC (class summary)	mCRPC post‑ARPI/chemo/RLT	PSMA‑high (IHC/PET)	Early‑phase single‑arm	ORR/PSA50	Activity limited in earlier constructs; optimization ongoing	Ocular/Skin with MMAE; neuropathy with DM1	Varied payloads; design re‑tooling	Inform sequencing with PSMA‑RLT	[59, 60]
STEAP1‑ADC (DSTP3086S)	mCRPC (early phase)	STEAP1 positive	Phase I single‑arm	ORR/PSA50/safety	Modest signals	Cytopenias, fatigue	MMAE (historical)	Programs re‑tooling	[61]
DLL3‑targeting ADCs (class summary)	NEPC/Small‑cell‑like PCa	DLL3 high in NEPC	Basket/Early phase (SCLC legacy informs NEPC)	ORR	Biology validated; legacy halted for toxicity	Effusions, cytopenias (legacy)	Re‑engineered designs in development	Rationale strong; trials re‑emerging	[62]
DS‑6000a (R‑DXd; CDH6/DXd)	ccRCC ± prior IO/TKI	CDH6 expressed in subsets	Phase I‐II single‑arm	ORR, DCR	Encouraging responses (early)	ILD/Pneumonitis, cytopenias, nausea	DXd bystander	Expansion ongoing; randomized planning	[63]

Abbreviations: ARPI, androgen receptor pathway inhibitor; ccRCC, clear‐cell renal cell carcinoma; DOR, duration of response; ILD, interstitial lung disease; IO, immune checkpoint inhibitor; la/mUC, locally advanced/metastatic urothelial carcinoma; mCRPC, metastatic castration‐resistant prostate cancer; ORR, objective response rate; OS, overall survival; PFS/rPFS, (radiographic) progression‐free survival; PN, peripheral neuropathy; PSA50, ≥50% PSA decline; RLT, radioligand therapy; Val‐Cit, valine‐citrulline.

### 4.1 UC: A Paradigm‐Setting Disease for ADC Therapy

UC represents the most clinically advanced setting for ADC development among genitourinary malignancies and one of the most successful examples across solid tumors. Several biological features contribute to this permissive landscape, including high epithelial antigen expression, relatively accessible tumor architecture, and pronounced intratumoral heterogeneity that can be exploited by bystander killing [64].

Nectin‐4‐directed ADCs have established the clinical proof‐of‐concept for this approach. The rapid adoption of enfortumab vedotin across multiple lines of therapy reflects a favorable alignment of target biology, efficient internalization, and a membrane‐permeable payload capable of overcoming spatial antigen heterogeneity. Importantly, clinical efficacy has been observed across a broad range of expression levels, underscoring that functional target suitability can outweigh strict quantitative thresholds [65].

The integration of ADCs into first‐line treatment has marked a major inflection point in UC management. Combination strategies pairing ADCs with immune checkpoint blockade have demonstrated that ADCs can function not only as cytotoxic agents but also as immune‐modulating therapies, capable of reshaping the tumor microenvironment and enhancing antitumor immunity. This shift from later‐line salvage therapy to frontline use represents a defining milestone for the ADC field as a whole [6].

Beyond nectin‐4, additional targets have expanded the ADC repertoire in UC. TROP2‐directed ADCs have shown activity in heavily pretreated disease, illustrating how broadly expressed epithelial antigens can be therapeutically leveraged when paired with appropriate payloads [66]. Similarly, HER2‐directed ADCs have redefined biomarker paradigms in UC, demonstrating clinically meaningful activity in tumors with low or heterogeneous HER2 expression. Collectively, these experiences highlight that ADC success in UC is driven by a combination of permissive biology, rational engineering, and flexible biomarker interpretation [67].

### 4.2 Prostate Cancer: Lineage Specificity Meets Phenotypic Plasticity

In contrast to UC, prostate cancer presents a more constrained and complex landscape for ADC development. Although several prostate‐associated antigens exhibit high lineage specificity, their clinical exploitation has been challenged by heterogeneous expression, dynamic regulation under androgen receptor signaling, and treatment‐induced phenotypic plasticity [68].

Prostate‐specific membrane antigen exemplifies both the promise and limitations of ADC targeting in this disease. Although it demonstrates robust internalization and high tumor specificity, early ADC programs were limited by narrow therapeutic windows and off‐tumor toxicity. The divergent clinical trajectories of PSMA‐directed ADCs and radioligand therapies underscore the importance of payload modality and exposure profile. Although radiopharmaceuticals benefit from sustained target engagement and spatially confined radiation, ADCs remain dependent on productive intracellular trafficking and lysosomal processing [69].

Additional prostate cancer targets, including STEAP1 and immune checkpoint‐related antigens, further illustrate the central challenge of phenotypic heterogeneity [57, 61]. As tumors progress to advanced and castration‐resistant states, lineage plasticity and neuroendocrine differentiation can attenuate antigen expression, undermining the durability of ADC responses. Moreover, the immune‐cold microenvironment and dense stromal components characteristic of advanced prostate cancer may further restrict payload penetration and immune‐mediated synergy [70].

Taken together, prostate cancer highlights a critical principle for ADC development: Lineage specificity alone is insufficient to guarantee clinical success. Durable activity requires alignment between target stability, payload properties, and combination strategies capable of counteracting adaptive resistance mechanisms.

### 4.3 RCC: Biological Constraints and Translational Challenges

RCC presents yet another distinct paradigm for ADC development, shaped by hypoxia‐driven transcriptional programs, pronounced intratumoral heterogeneity, and unique metabolic adaptations. Multiple lineage‐enriched antigens have been explored in RCC, particularly in clear‐cell disease, where constitutive hypoxia signaling drives target expression [71].

Despite strong biological rationale, historical ADC programs in RCC have achieved limited clinical success. Several factors contribute to this outcome, including inconsistent intracellular payload delivery, on‐target toxicity in normal tissues, and tumor microenvironmental features that constrain ADC penetration. Abundant vascularization, while theoretically favorable for drug delivery, does not necessarily translate into efficient intracellular payload accumulation, particularly when lysosomal processing is impaired or trafficking pathways are diverted [47].

Recent RCC‐focused ADC efforts have increasingly emphasized refined engineering strategies, including lower drug‐to‐antibody ratios, enhanced linker stability, and biomarker‐enriched trial designs. Although no ADC has yet achieved regulatory approval in RCC, ongoing early phase programs continue to generate valuable insights into target prioritization and patient selection. RCC thus reinforces the concept that antigen prevalence must be interpreted in the context of functional delivery competence and tolerable normal tissue expression.

### 4.4 Cross‐Cancer Comparison and Transferable Clinical Lessons

When viewed collectively, the uro‐oncological experience reveals several transferable principles relevant to ADC development across solid tumors. First, permissive tumor architecture and epithelial differentiation states, as seen in UC, favor ADC efficacy, particularly when paired with membrane‐permeable payloads. Second, phenotypic plasticity and dynamic antigen regulation, exemplified by prostate cancer, limit the durability of lineage‐restricted targeting strategies unless counterbalanced by rational combinations. Third, biological constraints on intracellular processing, as encountered in RCC, highlight the need for functional rather than purely expression‐based target evaluation [72, 73].

Importantly, these lessons resonate beyond uro‐oncology. Similar patterns have emerged in breast and lung cancers, where ADC efficacy depends on the interplay between antigen accessibility, payload biology, and treatment context rather than absolute expression thresholds. The uro‐oncological paradigm therefore provides a clinically grounded framework for understanding why certain ADC programs succeed, whereas others falter, informing future trial design, biomarker development, and therapeutic sequencing across oncology.

## 5 Biomarkers and Patient Selection for ADC Therapy

As ADCs move earlier in treatment paradigms and into combination regimens, biomarker‐driven patient selection has emerged as a central determinant of clinical success [30]. Unlike small‐molecule targeted therapies, for which discrete genomic alterations may serve as sufficient predictors of response, ADC activity is governed by a multidimensional set of biological and pharmacological variables [74]. These include not only tumor antigen expression, but also spatial heterogeneity, intracellular trafficking competence, payload susceptibility, and host‐specific factors influencing drug metabolism and tolerance [75]. Consequently, no single biomarker is likely to fully capture ADC sensitivity, necessitating integrated and dynamic selection strategies.

### 5.1 Antigen Expression: Beyond Binary Positivity

Tumor antigen expression remains the most established criterion for ADC eligibility, yet its predictive value is highly context dependent. Increasing evidence across solid tumors indicates that binary classification of antigen status is insufficient to reflect the biological complexity underlying ADC response [40]. Clinical activity has been observed across a continuum of expression levels for multiple ADC targets, challenging traditional paradigms derived from monoclonal antibody or small‐molecule development.

Quantitative assessment of antigen density provides a more nuanced framework for patient selection. Semiquantitative immunohistochemistry scoring systems and digital pathology platforms allow continuous rather than dichotomous evaluation of target expression. Importantly, moderate antigen expression may be sufficient for clinical efficacy when internalization is efficient and payload release is robust, whereas high expression does not guarantee response if downstream processing is impaired.

Spatial heterogeneity further complicates reliance on archival tissue samples [76]. Discordant antigen expression between primary tumors and metastases, as well as intralesional variability, can lead to sampling bias and misclassification. These limitations underscore the need to interpret antigen expression as a probabilistic rather than deterministic biomarker in the ADC context.

### 5.2 Functional Biomarkers: Internalization and Intracellular Processing

Beyond surface expression, functional suitability for ADC delivery has emerged as a critical yet underexplored dimension of biomarker development [77]. Productive ADC activity requires receptor‐mediated internalization, appropriate endocytic routing, and lysosomal environments capable of releasing the cytotoxic payload [78]. Differences in any of these steps can profoundly influence efficacy, even among tumors with comparable antigen density.

Preclinical and translational studies have demonstrated that altered endosomal trafficking, accelerated recycling of target‐ADC complexes, or impaired lysosomal maturation can attenuate payload release. Although direct clinical assays for these processes are not yet routinely available, emerging surrogate markers—including expression of endocytic regulators, lysosomal enzymes, and autophagy‐related pathways—may eventually complement antigen‐based selection [79].

The incorporation of functional biomarkers reflects a conceptual shift from static target identification toward dynamic evaluation of intracellular drug delivery competence. This shift is particularly relevant for tumors characterized by metabolic plasticity or adaptive stress responses, where intracellular processing pathways may be rewired under therapeutic pressure.

### 5.3 Liquid Biopsy and Dynamic Monitoring

Liquid biopsy approaches offer an attractive solution to the limitations of tissue‐based biomarker assessment. Circulating tumor DNA provides a noninvasive window into tumor burden, clonal evolution, and early molecular response, while circulating tumor cells and extracellular vesicles may convey information about antigen expression and phenotypic state [80].

In the context of ADC therapy, liquid biopsy is particularly valuable for longitudinal monitoring. Dynamic changes in antigen expression under selective pressure, early signals of response or resistance, and emergence of adaptive escape mechanisms may be detected prior to radiographic progression. Although clinical validation is ongoing, integration of liquid biopsy into ADC trials holds promise for adaptive treatment strategies, including early switching, sequencing decisions, and combination escalation [81] (Table 2).

**TABLE 2:** Multimodal biomarker toolkit for antibody–drug conjugate (ADC) patient selection and longitudinal monitoring: tissue, liquid biopsy, imaging, and functional assays.

Modality	Sample	Measures	Turnaround (days)	Threshold/Cut‐off (example)	Evidence level	Companion‑Dx readiness	Typical use case	Refs.
Quantitative IHC—Nectin‑4	FFPE tissue	*H*‑score or % positive	2–3	No mandatory cut‑off in UC trials	High (EV program)	High (approved drug; no companion Dx required)	Baseline selection; re‑biopsy for spatial/temporal heterogeneity	(EV‑302)/(EV‑201) [52, 82]
Quantitative IHC—Trop‑2	FFPE tissue	*H*‑score or % positive	2–3	Exploratory; no mandated cut‑off	Moderate (SG program)	Medium	Baseline characterization; correlative analyses	(TROPHY‑U‑01) [53]
Quantitative IHC—HER2	FFPE tissue	IHC 0/1+/2+/3+	2–3	IHC ≥1+ (HER2‑low included)	High/Moderate (DV trials)	Medium (assay harmonization ongoing)	Baseline selection; stratify HER2‑low vs. HER2‑high	(SGNDV‐001) [54]
Targeted PET—PSMA PET	Whole body	SUVmax/Uptake distribution	1–3	High uptake preferred	Moderate (extensive PCa data)	Medium	Dose individualization; early futility in low‑uptake disease	[83, 84]
ImmunoPET—HER2 (e.g., 89Zr‑trastuzumab)	Whole body	Target expression imaging	1–3	No unified cut‑off	Emerging	Low–medium	Map heterogeneity; predict early non‑response	[85, 86]
ctDNA	Plasma	Variant allele fraction; DDR/TOP1 pathways; tumor fraction	5–7	Molecular response: ≥50% VAF drop by Weeks 6–8	High–moderate	Medium	Early molecular response (Weeks 6–8) go/no‑go; resistance pathway readouts	(NCT04964934)/(NCT04354064) [87]
Exosomal proteins	Urine/Plasma	Serial antigen abundance (ELiza/EV‑capture)	3–7	Relative change vs. baseline ≥30%–50%	Emerging‐moderate	Low–medium	On‑treatment adaptation (C2–C3); flag antigen loss or surge	[88, 89]
AI‑assisted digital pathology	FFPE tissue	Automated scoring; heterogeneity indices	1–2	Agreement with human reads: kappa ≥0.7	Emerging	Low–medium	Standardize reads; quantify heterogeneity for eligibility	[90, 91]
Spatial transcriptomics/Multiplex RNA‑ISH	FFPE/Fresh	Antigen mRNA + trafficking/endocytosis markers	7–10	Quantitative cut‑offs under exploration	Emerging	Low	Heterogeneity mapping; bystander‑effect inference; research/assay development	[92, 93]
Ex vivo internalization assays	Fresh tissue/Organoids	Internalization rate; recycling vs. lysosomal routing	5–10	Association of internalization half‑life with ADC activity (exploratory)	Exploratory	Low	Target‐payload matching; preclinical‑to‑clinical bridging	[94, 95, 96]

Abbreviations: ctDNA, circulating tumor DNA; DDR, DNA damage response; EV, extracellular vesicle; FFPE, formalin‐fixed paraffin‐embedded; IHC, immunohistochemistry; ImmunoPET, antibody‐based PET imaging; PET, positron emission tomography; RNA‐ISH, RNA in situ hybridization; SUVmax, maximum standardized uptake value.

### 5.4 Molecular Imaging and Spatial Pharmacodynamics

Molecular imaging represents a complementary modality for whole‐body assessment of target expression and ADC biodistribution [97]. Antibody‐based positron emission tomography tracers and radiolabeled surrogates enable noninvasive evaluation of antigen distribution across primary and metastatic sites, capturing spatial heterogeneity that cannot be reliably assessed through biopsy alone [98].

Beyond target detection, imaging‐based approaches may provide pharmacodynamic insights into tumor uptake and retention, linking ADC engineering properties to in vivo behavior. In tumors with multifocal or heterogeneous metastatic patterns, molecular imaging may identify discordant lesions that limit overall treatment efficacy and guide more informed patient selection [99].

### 5.5 Host Factors and Pharmacogenomic Determinants

Host‐specific variables also contribute substantially to interindividual variability in ADC exposure and toxicity. Pharmacogenomic differences affecting payload metabolism can influence systemic drug levels and adverse event risk, particularly for ADCs carrying topoisomerase I inhibitor payloads. Baseline organ function, prior treatment history, and inflammatory status further modulate tolerability and may necessitate dose adjustment or alternative sequencing strategies.

Recognition of these host factors is essential for preserving dose intensity and optimizing therapeutic index, especially as ADCs are combined with immune checkpoint inhibitors or other cytotoxic agents. Incorporating pharmacogenomic and clinical parameters into patient selection frameworks represents an important step toward personalized ADC therapy [100].

### 5.6 Toward Integrated and Adaptive Biomarker Frameworks

Collectively, these considerations underscore that effective patient selection for ADC therapy requires a multimodal biomarker strategy. Integrating quantitative and spatial tissue profiling, functional assessments of intracellular processing, liquid biopsy‐based monitoring, molecular imaging, and host‐specific variables offers a path toward more precise and dynamic stratification.

Uro‐oncological malignancies, with their well‐characterized antigen landscapes and expanding clinical ADC experience, provide a fertile setting for developing and validating such integrated frameworks. As ADC platforms continue to evolve, biomarker strategies must co‐develop with engineering advances, enabling rational patient selection, optimized sequencing, and adaptive treatment approaches across oncology.

## 6 Mechanisms of Resistance to ADCs

**FIGURE 3:**
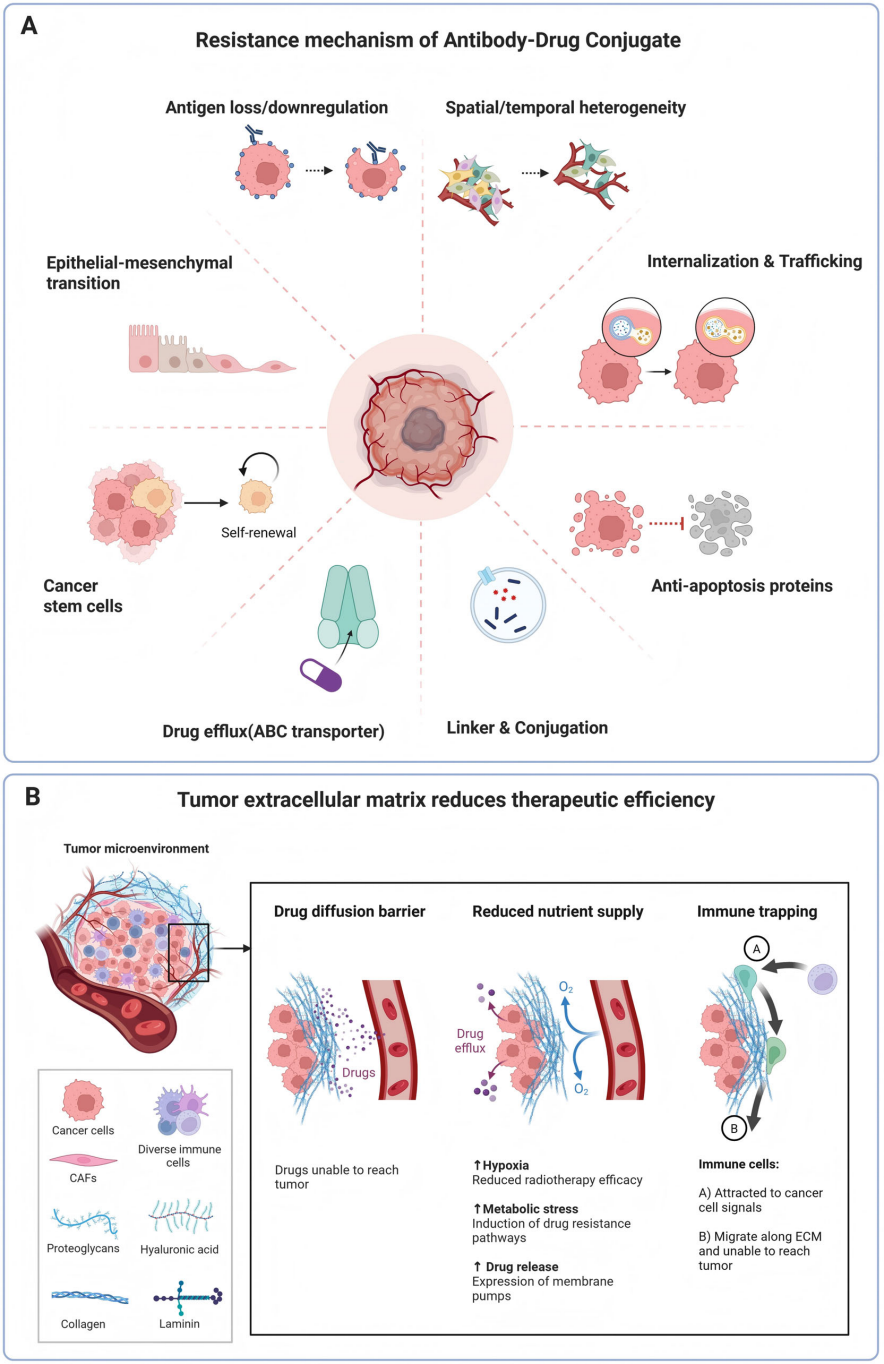
Mechanisms of resistance to ADCs and matrix‐mediated barriers. (A) Tumor‐intrinsic and target‐related mechanisms of resistance to ADCs, including antigen loss or heterogeneity, impaired internalization or lysosomal trafficking, linker or conjugation liabilities, activation of pro‐survival signaling, drug efflux, and lineage plasticity. (B) Extracellular matrix‐mediated barriers to ADC efficacy, in which desmoplastic stroma and abnormal vasculature restrict tumor penetration, promote hypoxia and metabolic stress, and attenuate both payload delivery and immune‐mediated effects. ABC, ATP‐binding cassette; CAFs, cancer‐associated fibroblasts; DAR, drug‐antibody ratio; ECM, extracellular matrix; EMT, epithelial‐mesenchymal transition.

Despite the growing clinical success of ADCs, both primary and acquired resistance remain major barriers to durable therapeutic benefit across solid tumors [101]. Unlike resistance to small‐molecule targeted therapies, which is often driven by discrete genetic alterations, ADC resistance is typically multifactorial and reflects the composite nature of these agents [102]. Resistance mechanisms may emerge at multiple levels, encompassing target biology, intracellular trafficking, payload handling, and tumor microenvironmental adaptation. Understanding these layered processes is essential for informing next‐generation ADC design, biomarker development, and rational combination strategies [4] (Figure 3).

### 6.1 Target Modulation and Antigen Heterogeneity

Alterations in target antigen expression represent one of the most intuitive and clinically relevant mechanisms of ADC resistance [103]. Downregulation, heterogeneous loss, or lineage switching of antigen‐positive tumor cell populations can reduce ADC binding and internalization, thereby limiting effective payload delivery. Importantly, resistance does not require complete antigen loss; even partial or spatially restricted downregulation may suffice to compromise efficacy, particularly when bystander killing is limited.

Dynamic antigen modulation under therapeutic pressure is well documented in several solid tumors. Treatment‐induced phenotypic plasticity, epithelial‐mesenchymal transition, and lineage transdifferentiation can all attenuate target expression over time. In this context, baseline antigen positivity should be viewed as a conditional rather than permanent state, highlighting the importance of longitudinal monitoring and adaptive treatment strategies.

### 6.2 Impaired Internalization and Intracellular Trafficking

Preserved antigen expression does not guarantee productive ADC activity. Resistance may arise from alterations in receptor‐mediated internalization or intracellular routing that divert ADCs away from lysosomal compartments required for payload release. Accelerated recycling of antigen‐ADC complexes back to the cell surface, altered endosomal maturation, or diversion toward non‐degradative pathways can markedly reduce intracellular drug exposure [104].

Defects in lysosomal function further contribute to resistance. Altered vesicular pH, reduced protease activity, or enhanced autophagic flux may impair linker cleavage or antibody degradation, attenuating payload liberation. These mechanisms are particularly relevant in tumors with high metabolic adaptability, where intracellular trafficking pathways can be rewired in response to therapeutic stress [79]. Collectively, these observations underscore that intracellular processing competence is a critical determinant of sustained ADC sensitivity.

### 6.3 Payload Efflux and Downstream Drug Resistance

Resistance mechanisms downstream of payload release add an additional layer of complexity [105]. Upregulation of drug efflux transporters, particularly ATP‐binding cassette family proteins, can actively export released payloads, reducing intracellular concentrations below cytotoxic thresholds. This phenomenon has been described for both microtubule inhibitors and topoisomerase I inhibitor payloads and may preferentially affect ADCs with membrane‐permeable warheads [106].

Tumor‐intrinsic alterations in drug response pathways further modulate payload sensitivity. Enhanced DNA damage repair capacity, replication stress tolerance, or alterations in microtubule dynamics can blunt the cytotoxic effects of ADC payloads. While these mechanisms parallel resistance observed with corresponding free drugs, the kinetics and spatial distribution of payload exposure unique to ADCs may shape distinct resistance trajectories [107].

### 6.4 Tumor Microenvironment‐Mediated Resistance

The tumor microenvironment exerts a profound influence on ADC efficacy and resistance. Dense stromal architecture, abnormal vasculature, and hypoxic niches can impede ADC penetration and create spatial gradients of drug exposure within tumors. In immune‐suppressed or immune‐excluded tumors, limited engagement of immune effector mechanisms may further constrain the contribution of Fc‐mediated activity and immunogenic cell death [108].

In addition, nonmalignant cells within the microenvironment may sequester ADCs through Fc receptor‐mediated uptake, reducing the fraction of drug reaching tumor cells. Macrophage‐mediated clearance and extracellular matrix remodeling can therefore indirectly contribute to therapeutic resistance [109]. These barriers are particularly relevant in tumors with prominent stromal components, where physical and biological constraints converge to limit payload delivery.

### 6.5 Clinical Implications and Strategies to Overcome Resistance

The multifactorial nature of ADC resistance suggests that no single intervention is likely to be universally effective. Instead, resistance mechanisms map directly onto actionable strategies at the levels of engineering, biomarker‐guided patient selection, and rational combination therapy.

From an engineering perspective, enhancing bystander effect through payload permeability, optimizing linker chemistry to ensure efficient intracellular release, and selecting targets with durable expression profiles may mitigate upstream resistance. Incorporating controlled‐release or conditionally activated payload systems offers additional opportunities to overcome intracellular processing constraints [110].

Combination strategies provide a complementary approach to counteract resistance. Pairing ADCs with immune checkpoint inhibitors may amplify immunogenic cell death and remodel immune‐suppressive microenvironments, whereas combinations with DNA damage response inhibitors, epigenetic modulators, or agents targeting intracellular trafficking pathways may resensitize tumors to payload‐induced cytotoxicity. Importantly, these approaches must be balanced against cumulative toxicity, reinforcing the need for biomarker‐informed combination design.

### 6.6 Resistance as a Driver of Next‐Generation ADC Innovation

Rather than representing a terminal limitation, resistance to ADCs has increasingly become a driver of innovation. Insights into antigen modulation, trafficking escape, and payload resistance are actively informing the design of next‐generation ADC platforms, including bispecific constructs, alternative payload classes, and tumor‐restricted activation strategies.

Integrating resistance biology with real‐time biomarker monitoring and adaptive trial designs will be essential for extending the durability of ADC responses. In this regard, resistance should be viewed not merely as a clinical obstacle, but as a roadmap guiding the evolution of ADC therapy across oncology.

## 7 Combination Strategies and Clinical Integration

**FIGURE 4:**
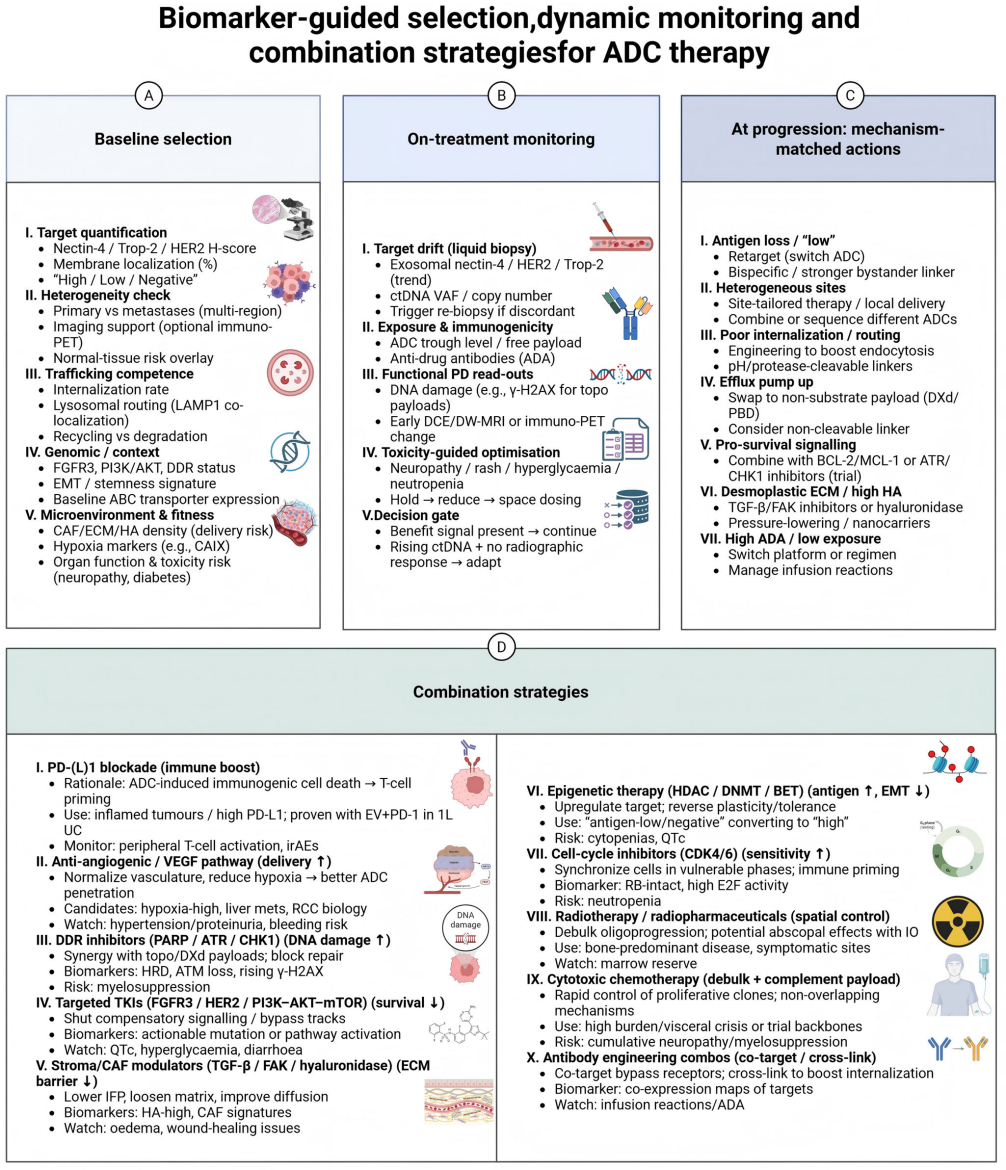
Integrated selection, monitoring, and adaptive strategies for ADC therapy. (A) Baseline patient selection using multimodal profiling, incorporating quantitative and spatial assessment of target expression, trafficking competence, tumor genomic context, microenvironmental features, and host factors to inform ADC choice and dosing. (B) On‐treatment monitoring using serial liquid biopsy, pharmacokinetic and pharmacodynamic read‐outs, functional imaging, and toxicity‐guided optimization to distinguish continued benefit from early futility. (C) Mechanism‐matched adaptive strategies at progression, including retargeting, payload or linker switching, engineering‐based solutions to trafficking defects, and biologically rational combinations. (D) Goal‐directed combination strategies organized by primary objective, such as immune activation, enhancement of payload delivery, amplification of DNA damage, suppression of survival signaling, reduction of stromal barriers, or optimization of treatment sequencing. ctDNA, circulating tumor DNA; DDR, DNA‐damage response; EV, enfortumab vedotin; HA, hyaluronan; UC, urothelial carcinoma.

As ADCs move from later‐line settings into earlier treatment paradigms, combination strategies have become central to extending response durability and maximizing clinical benefit [111, 112]. Unlike conventional chemotherapy, ADCs introduce unique opportunities for rational combination design, owing to their target specificity, payload‐dependent mechanisms of action, and potential immunomodulatory effects [25, 113, 114]. At the same time, these features impose distinct challenges related to overlapping toxicity, sequencing, and cumulative exposure. Effective integration of ADCs into combination regimens therefore requires careful alignment of biological rationale with clinical feasibility (Figure 4).

### 7.1 ADCs Combined With Immune Checkpoint Blockade

The combination of ADCs with immune checkpoint inhibitors represents the most clinically mature and biologically compelling strategy to date. Several ADC payload classes, particularly microtubule inhibitors and topoisomerase I inhibitors, can induce immunogenic cell death, enhance antigen presentation, and promote immune cell infiltration within the tumor microenvironment. These effects provide a strong mechanistic rationale for synergy with immune checkpoint blockade. Increasingly, big data‐driven immunotherapy frameworks provide a practical route to prioritize immunology for rational ADC combinations. The Cancer Immunology Data Engine (CIDE) is a large‐scale clinical trial framework that integrates multi‐omics tumor profiles with ICB and T‐cell therapy outcomes. Anchored on trial outcomes, CIDE has identified candidates synergized with ICBs, including AOAH, CR1L, COLQ, ADAMTS7, AGR2, and CDK10 [115]. These candidates can be leveraged to design mechanism‐informed ADC regimens combined with ICBs (PD‐L1, PD‐1, B7‐H3, CTLA4) or tumor antigens (HER2, GPC3, GD2) to induce immune‐priming effects for long‐term responses compared to conventional cytotoxic ADCs.

Clinical experience across solid tumors has demonstrated that ADCs can function as immune‐priming agents rather than purely cytotoxic drugs. In uro‐oncological malignancies, this paradigm has been most clearly illustrated in UC, where ADC‐ICI combinations have reshaped frontline treatment strategies. Importantly, the observed synergy likely reflects a convergence of mechanisms, including payload‐induced tumor cell death, Fc‐mediated immune engagement, and bystander killing that amplifies antigen release. However, ADC‐ICI combinations also introduce overlapping toxicity profiles, particularly dermatologic and gastrointestinal adverse events [116]. Successful implementation therefore depends on optimized dosing, vigilant monitoring, and early intervention strategies to preserve dose intensity without compromising safety [114].

### 7.2 Targeting DNA Damage Response and Cell‐Cycle Pathways

Given that several widely used ADC payloads exert their cytotoxic effects through DNA damage or replication stress, combinations with DNA damage response inhibitors offer a rational strategy to enhance efficacy and overcome resistance [117]. Inhibition of key repair pathways can potentiate payload‐induced cytotoxicity by preventing tumor cells from resolving DNA lesions.

This approach is particularly attractive in tumors with pre‐existing defects in DNA repair or high proliferative stress, where ADC payloads may already exert selective pressure. Nevertheless, the risk of cumulative myelosuppression represents a major clinical limitation. Rational scheduling, biomarker‐guided patient selection, and careful dose optimization are therefore essential to avoid unacceptable toxicity [78].

### 7.3 Modulating the Tumor Microenvironment

The tumor microenvironment plays a dual role in ADC therapy, acting both as a barrier to effective drug delivery and as a potential target for therapeutic synergy. Stromal density, myeloid cell infiltration, and immune exclusion can all limit ADC penetration and attenuate immune‐mediated effects [118].

Combinations that modulate the tumor microenvironment may enhance ADC efficacy by improving payload distribution or relieving immune suppression [119]. Strategies targeting tumor‐associated macrophages, stromal components, or inflammatory signaling pathways hold particular promise in tumors characterized by dense stroma or immunosuppressive niches. Importantly, such approaches should be tailored to tumor‐specific microenvironmental features rather than applied uniformly, reinforcing the need for biomarker‐informed combination design.

### 7.4 ADCs With Radiopharmaceuticals and Targeted Radiation

Radiopharmaceutical therapies introduce a complementary mode of targeted cytotoxicity that may synergize with ADCs through distinct but convergent mechanisms. Although both modalities rely on target expression, their intracellular requirements differ substantially. Radioligands deliver cytotoxic radiation independent of lysosomal processing, whereas ADCs depend on intracellular trafficking and payload release [37].

This divergence raises the possibility that sequential or carefully timed combination strategies could overcome resistance arising from impaired ADC internalization or lysosomal dysfunction. In prostate cancer, where radioligand therapy has demonstrated clear clinical benefit, integration with ADCs represents an area of emerging interest. However, overlapping marrow toxicity remains a key concern, favoring sequential rather than concurrent administration in many clinical scenarios [120].

### 7.5 Metabolic and Signaling Pathway Combinations

Tumor metabolic state and oncogenic signaling pathways can influence ADC sensitivity by modulating intracellular trafficking, lysosomal function, and stress response pathways. Adaptive metabolic reprogramming may enable tumor cells to tolerate payload‐induced stress, contributing to resistance.

Targeting metabolic dependencies or adaptive signaling networks therefore represents a potential strategy to resensitize tumors to ADC therapy. Such combinations remain largely exploratory but may be particularly relevant in tumors with pronounced metabolic plasticity, including RCC and advanced prostate cancer. As with other combination strategies, robust mechanistic rationale and biomarker support are essential to justify clinical translation.

### 7.6 Principles for Clinical Integration and Sequencing

Beyond biological synergy, successful combination strategies must account for clinical integration and treatment sequencing. Prior exposure to ADCs, chemotherapy, immune checkpoint inhibitors, or radiopharmaceuticals can influence subsequent tolerability and efficacy. Thoughtful sequencing strategies are therefore required to maximize cumulative benefit while preserving future treatment options.

Importantly, ADC combinations should be viewed as programmatic rather than opportunistic. Selection of combination partners, timing of administration, and toxicity management must be considered holistically, guided by both tumor biology and patient‐specific factors. As ADCs move earlier in the disease course, these considerations will become increasingly central to long‐term patient outcomes.

## 8 Challenges in Clinical Development and Implementation

As ADCs become integrated into earlier treatment lines and increasingly complex combination regimens, challenges in clinical development and real‐world implementation have moved to the forefront [121]. Although ADCs are designed to improve tumor selectivity, their clinical use introduces distinct safety, logistical, and sequencing considerations that differ fundamentally from those of conventional chemotherapy or targeted therapies. Addressing these challenges in a systematic manner is essential for preserving dose intensity, maximizing cumulative benefit, and ensuring sustainable clinical adoption [122, 123] (Table 3).

**TABLE 3:** Payload‐driven toxicity fingerprints of antibody–drug conjugates (ADCs) and a practical, grade‐based management framework.

Payload/Class	Representative agents	Key AEs (≥Grade 3 focus)	Typical onset window	Major risk factors	Baseline and on‑treatment monitoring	Prophylaxis/Early mitigation	Grade‑based management	Standard dose mods (examples)	Re‑challenge rules	Refs./Registry
MMAE (microtubule inhibitor; bystander‑capable)	Enfortumab vedotin, Disitamab vedotin, FOR46, STEAP1‑ADC	Dermatologic rash, peripheral neuropathy, hyperglycemia	Rash/hyperglycemia: cycles 1‐2; PN cumulative (often ≥Cycle 3)	Pre‑existing PN, diabetes, neurotoxic co‑medications	Skin exam; neurologic exam; FPG/HbA1c; CBC/LFTs	Skin care and sun protection; early topical/systemic steroids; optimize glucose	PN: Grade 2 → hold until ≤G1 then reduce dose; Grade ≥3 → discontinue. Rash: Grade 2 → topical ± short systemic steroids and consider delay; Grade ≥3 → hold/consider discontinue. Hyperglycemia: glucose ≥250–300 mg/dL → hold, insulin, correct dehydration/infection	EV example: 1.25 → 1.0 → 0.75 mg/kg (D1, 8, 15 q28d); DV similar per PI	Re‑start when PN/rash resolve to ≤G1 and stable; avoid re‑challenge after severe recurrence	[7, 124, 125, 126, 127, 128]
DXd (topoisomerase‑I; ILD vigilance)	Ifinatamab deruxtecan (DS‑7300), DS‑6000a (R‑DXd)	Interstitial lung disease/pneumonitis, neutropenia, nausea	ILD often within first 3–6 months; neutropenia cycles 1–2	Prior ILD or chest RT, lung disease, smoking, older age, low BMI	Baseline chest imaging and SpO_2_; symptom checks each visit; CBC	Patient education on cough/dyspnea; antiemetics; avoid lung‑toxic co‑meds	Any suspected ILD: hold immediately and start systemic steroids; Grade 1 confirmed → consider dose reduction and cautious resume; Grade ≥2 → permanently discontinue. Manage neutropenia per CTCAE with G‑CSF support as needed	Typical reduction: 6.4 → 5.4 → 4.4 mg/kg (molecule‑specific)	Resume only after complete resolution and pulmonology clearance (Grade 1 only); Grade ≥2 do not resume	[129, 130, 131]
SN‑38/topoisomerase‑I	Sacituzumab govitecan	Neutropenia, late diarrhea, nausea/vomiting, alopecia	Common in Cycles 1–2; late diarrhea often >24 h post‑dose	UGT1A1*28/*6 genotype, hepatic impairment, heavy prior therapy, older age	CBC with differential; electrolytes; hydration status; temperature; diarrhea diary	Early diarrhea: atropine; late diarrhea: loperamide protocol plus fluids/electrolytes; consider prophylactic G‑CSF	Grade 3 neutropenia: hold, G‑CSF, resume at reduced dose; febrile neutropenia: hospitalize and broad‑spectrum antibiotics; Grade ≥3 diarrhea: IV fluids/electrolytes/antidiarrheals, consider admission	Common reductions: 10 → 7.5 → 5 mg/kg (D1, 8 q21d) per PI	Resume when ≤G1 and stable without infection; repeated Grade ≥3 may warrant discontinuation	[132, 133, 134, 135]
Duocarmycin (DNA alkylator)	MGC018 (B7‑H3)	Myelosuppression, fatigue, nausea	Usually Cycles 1–2	Low marrow reserve, prior chemoradiation	CBC; symptom checks	Supportive care; G‑CSF and antiemetics as needed	Adjust/hold per CTCAE; consider discontinuation for persistent Grade ≥3	Protocol‑specific reductions	Resume when ≤G1; consider termination for recurrent severe toxicity	[136, 137]
Infusion reactions/hypersensitivity (payload‑agnostic)	All ADCs	Infusion reaction, hypersensitivity/anaphylaxis	Typically first or early infusions	Prior allergies, rapid infusion rate	Vital signs; rash/wheezing monitoring	Premedication with antipyretic/antihistamine ± steroid; slower infusion rate	Mild‐moderate: slow or interrupt infusion and treat symptoms; severe: stop infusion and manage per ACLS/acute care	Follow PI for first‑dose and re‑challenge strategies	Re‑challenge after resolution for mild‐moderate reactions; do not re‑challenge after anaphylaxis	[128, 138, 139, 140]

*Note*: Safety definitions: Adverse event grading follows CTCAE (unless otherwise specified). For suspected drug‐related ILD/pneumonitis, prompt drug interruption and early corticosteroid initiation are recommended; re‐challenge should be considered only for fully resolved Grade 1 events with specialist input, whereas Grade ≥2 generally warrants permanent discontinuation (agent‐ and label‐dependent).

Abbreviations: AE, adverse event; CBC, complete blood count; CTCAE, Common Terminology Criteria for Adverse Events; DXd, deruxtecan (topoisomerase‐I inhibitor payload); FPG, fasting plasma glucose; G‐CSF, granulocyte colony‐stimulating factor; HbA1c, glycated hemoglobin; ILD, interstitial lung disease; LFT, liver function test; MMAE, monomethyl auristatin E; PI/SmPC, prescribing information/summary of product characteristics; PN, peripheral neuropathy; RT, radiotherapy; SN‐38, active metabolite of irinotecan; SpO_2_, peripheral oxygen saturation.

### 8.1 Payload‐Driven Toxicity Fingerprints

The toxicity profiles of ADCs are largely determined by their payload classes rather than target expression alone. As a result, distinct and predictable “toxicity fingerprints” have emerged across clinically established ADC platforms [141]. Recognizing these patterns enables proactive monitoring and early intervention, which are critical to maintaining treatment continuity.

Auristatin‐based ADCs are commonly associated with peripheral neuropathy, dermatologic toxicity, and metabolic disturbances. These adverse events reflect both payload mechanism and low‐level target expression in normal tissues [142]. Topoisomerase I inhibitor‐based ADCs exhibit toxicity profiles dominated by myelosuppression and gastrointestinal effects, mirroring those of their free‐drug counterparts but modulated by ADC‐specific pharmacokinetics. In addition, certain ADC platforms introduce unique safety considerations, such as interstitial lung disease observed with some HER2‐directed constructs, necessitating heightened vigilance [143, 144].

Importantly, these toxicity patterns are not idiosyncratic but reproducible, underscoring that ADC safety can be anticipated and managed through agent‐specific frameworks rather than reactive dose modification alone.

### 8.2 Proactive Toxicity Management Frameworks

Effective ADC delivery requires a shift from reactive toxicity management toward structured, anticipatory supportive care. Increasingly, institutions have adopted multidisciplinary toxicity management pathways that integrate oncology, dermatology, neurology, pulmonology, and supportive care expertise [145, 146].

Key elements of such frameworks include baseline assessment of comorbidities relevant to payload‐specific risks, standardized patient education regarding early symptom recognition, and protocol‐driven monitoring schedules. Early intervention—such as topical or systemic therapy for dermatologic toxicity, prompt dose modification for neuropathy, or growth factor support for myelosuppression—can prevent progression to dose‐limiting severity and preserve treatment intensity [147].

Digital symptom monitoring tools and patient‐reported outcome platforms further enhance early detection, particularly as ADCs are administered in outpatient settings and combined with other systemic therapies [148]. These approaches are increasingly important as ADC use expands beyond specialized centers into broader clinical practice.

### 8.3 Treatment Sequencing and Cumulative Exposure

Treatment sequencing represents a critical but underexplored dimension of ADC implementation. Prior exposure to ADCs, chemotherapy, immune checkpoint inhibitors, or radiopharmaceuticals can influence subsequent tolerability and efficacy through cumulative toxicity, antigen modulation, or overlapping mechanisms of resistance.

As ADCs move earlier in the disease course, thoughtful sequencing strategies are required to maximize cumulative benefit across lines of therapy. Decisions regarding whether to sequence ADCs by target, payload class, or combination partner should be guided by both biological rationale and patient‐specific factors, including prior toxicity profiles and residual organ reserve. Importantly, inappropriate sequencing may compromise future treatment options by exhausting tolerability rather than tumor sensitivity.

### 8.4 Trial Design, Endpoints, and Translational Integration

Clinical trial design poses additional challenges for ADC development. Traditional endpoints such as objective response rate and progression‐free survival may not fully capture the clinical value of ADCs, particularly when bystander effects or lesion‐level heterogeneity contribute to mixed responses.

Incorporation of translational endpoints—including serial biomarker assessment, pharmacodynamic imaging, and patient‐reported outcomes—can provide a more comprehensive evaluation of ADC activity and tolerability. Adaptive trial designs that allow for biomarker‐driven enrichment or early treatment modification may further improve efficiency and translational relevance.

Close integration between clinical and translational research is particularly important for ADCs, given their composite nature and the need to link engineering features with in vivo behavior. Lessons learned from early phase trials should directly inform subsequent platform optimization and combination strategies.

### 8.5 Health‐System Readiness and Real‐World Implementation

Beyond biological and clinical considerations, sustainable ADC implementation depends on health‐system readiness. Manufacturing complexity, cost considerations, infusion logistics, and access to specialized supportive care all influence real‐world adoption [149].

As ADCs become standard components of first‐line and combination regimens, coordination across multidisciplinary teams and healthcare infrastructures will be essential. Education of clinicians and patients, development of standardized management pathways, and incorporation of real‐world evidence into post‐marketing evaluation will play increasingly important roles in optimizing outcomes at scale.

## 9 Conclusions and Future Perspectives

ADCs have transitioned from experimental constructs to a central pillar of contemporary oncology, driven by convergent advances in antibody engineering, linker chemistry, payload design, and clinical strategy [150]. Clinical successes across multiple solid tumors demonstrate that ADCs are no longer niche therapeutics but versatile platforms capable of reshaping treatment paradigms when biological insight, engineering optimization, and clinical execution are properly aligned [151].

Experience from uro‐oncological malignancies has been particularly instructive in this evolution. UC has emerged as a paradigm‐setting disease, illustrating how permissive tumor architecture, epithelial antigen expression, and bystander‐capable payloads can enable transformative clinical benefit [152]. In contrast, prostate and renal cancers highlight the biological constraints that limit ADC durability, including lineage plasticity, intracellular trafficking barriers, and microenvironmental resistance. Together, these malignancies underscore a central principle that extends across oncology: ADC success is not dictated by antigen prevalence alone, but by the functional integration of target biology, payload properties, and treatment context [153].

Looking forward, the future development of ADCs will increasingly be shaped by innovations that transcend first‐generation design principles. Advances in site‐specific conjugation, controlled‐release linkers, and conditionally activated payloads offer opportunities to expand the therapeutic index of highly potent warheads [154, 155]. Bispecific and multifunctional ADC constructs may further enhance tumor selectivity, facilitate productive intracellular routing, or engage complementary immune mechanisms. Importantly, these engineering advances are likely to be informed not by theoretical optimization alone, but by resistance mechanisms observed in clinical practice.

Biomarker strategies will play an equally critical role in shaping next‐generation ADC implementation. Static, binary assessments of antigen expression are giving way to integrated frameworks that combine quantitative and spatial tissue profiling, functional evaluation of intracellular processing, liquid biopsy‐based monitoring, and molecular imaging [156]. Such approaches will be essential for dynamic patient selection, rational sequencing, and early identification of resistance, particularly as ADCs move into earlier treatment lines and combination regimens [157].

Clinical development paradigms must also evolve to keep pace with ADC innovation. Adaptive trial designs, incorporation of translational endpoints, and systematic evaluation of combination strategies will be required to fully realize the potential of ADC platforms. Equally important is the development of structured toxicity management and implementation frameworks that enable safe, scalable adoption in real‐world practice [158, 159].

Ultimately, the sustainable success of ADCs will depend on their integration into a broader precision oncology ecosystem—one that aligns molecular engineering with tumor biology, biomarker science, and patient‐centered clinical care. Lessons learned from uro‐oncological malignancies provide a clinically grounded roadmap for this integration. As ADC technologies continue to mature, these insights will inform the rational expansion of ADC therapy across solid tumors, guiding the field toward more precise, durable, and effective cancer treatment.

## Author Contributions

Bisheng Cheng, Lanqi Gong, and Zongwei Wang contributed equally to this work. Bisheng Cheng, Lanqi Gong, and Zongwei Wang conceived the scope of the review, performed the literature search, and drafted the manuscript. Peidan Peng assisted with data extraction, figure/table preparation, and content organization. Kewei Xu and Hai Huang provided critical input on the structure and scientific content and revised the manuscript for important intellectual content. Peng Wu supervised the project, coordinated the writing process, and finalized the manuscript. All authors reviewed and approved the final version of the manuscript.

## Funding

This work was supported by the National Natural Science Foundation of China (82503289), President Foundation of Nanfang Hospital, Southern Medical University (2024A028), China Postdoctoral Science Foundation (2025M782052) to Bisheng Cheng. National Natural Science Foundation of China (82570912, 81870522, and 82173304), Guangzhou Key Research and Development Program (2023B03J1245) to P.W., National Natural Science Foundation of China (82472902), and Guangdong Provincial Key Research and Development Program (2023B1111030006) to H.H.

## Ethics Statement

The authors have nothing to report.

## Conflicts of Interest

The authors declare no conflicts of interest.

## Data Availability

The authors have nothing to report.
